# Prevalence of Bovine Schistosomiasis and Associated Risk Factors in Tis Abay District, Northwest Ethiopia

**DOI:** 10.1155/2022/8940576

**Published:** 2022-11-28

**Authors:** Temesgen Kifle, Tirumengist Bayile, Haben Fesseha, Mesfin Mathewos

**Affiliations:** School of Veterinary Medicine, Wolaita Sodo University, Wolaita Sodo, P.O. Box-t138, Ethiopia

## Abstract

**Background:**

Schistosomiasis is a parasitic disease of cattle that is caused by trematode worms and results in morbidity, mortality, reduced fertility, and productivity.

**Methods:**

A cross-sectional study was conducted to determine the prevalence and associated risk factors for bovine schistosomiasis in Tis Abay district, Amhara, Ethiopia. Fecal samples were collected from 384 randomly selected cattle and examined using the sedimentation technique.

**Results:**

Of the total examined fecal samples, 13.02% (50/384) of the samples were positive for *Schistosoma bovis* eggs. The prevalence of schistosomiasis was highly reported in Dasra (22.4%), which was statistically significant (*p* ≤ 0.001). The prevalence of bovine schistosomiasis was higher in females (16.9%), crossbred cattle (17.1%), poorly conditioned cattle (37.1%), extensively managed cattle (17.9%), and cattle greater than 5 years old (23.1%). The multivariate analysis of factors revealed that study area, age, breed, body condition, and management system have a significant role (*p* < 0.05) in the prevalence of bovine schistosomiasis.

**Conclusion:**

Schistosoma infection is a problem for cattle in the study region. Therefore, farmers should be aware of the transmission of the disease, prevention, and control of snails.

## 1. Introduction

Ethiopia has one of the largest livestock herds in Africa with an estimated of 70 million cattle, about 42.9 million sheep, 52.5 million goats, 2.15 million horses, 10.80 million donkeys, 0.38 million mules, about 8.1 million camels and 48.9 million Poultry [1]. However, cattle production and productivity are far below the expected potential due to widespread animal diseases, inadequate and poor-quality animal feed, limited veterinary services, poor selection and breeding, and lack of proper technology packages. Among the bottlenecks in cattle production, tapeworm parasites are predominant [2, 3]. Ruminant flukes are parasitic flatworms (flukes) that live in the liver (Fasciola), proventriculus (Paramphistomum), or blood (Schistosoma) [4].

Schistosomiasis is a parasitic disease caused by the genus Schistosoma and several types of species, including *Schistosoma bovis*, *S*. *indicum*, *S*. *japonicum*, *S*. *matthei*, *S*. *intercalatum*, *S*. *nasale*, and *S*. *rodhoni*. *S*. *bovis*, *S*. *matthei*, and *S*. *intercalatum* are the main species that cause schistosomiasis in ruminants. Parasitism is a major development challenge. Fluke infestation is one of the major problems limiting the productivity of both animals and humans worldwide [5].

Environmental factors such as moisture, rainfall, temperature, water bodies (stagnant ponds, swamps, streams, rivers, irrigation canals, marshes, and dams), and snail intermediary hosts influence both animal and human schistosomiasis [6]. Furthermore, Schistosoma infection is linked to infected water sources with conventional grazing and watering systems [7]. These parasites are found in large water-logged and marshy grazing fields, which is expected to be optimal for the propagation and maintenance of the intermediate host (snails) and thus the high prevalence of trematode infection [8].

Clinical indicators, seasonal occurrence in endemic locations, agroecology of the area or identification of snail habitats, postmortem examinations, and inspection of feces for fluke eggs are used to diagnose schistosomiasis; however, coprological analysis is usually utilized. Schistosomiasis treatment aids in the reversal of acute or early chronic disease, as well as the prevention of problems associated with chronic infection. The most efficient strategy to control cattle schistosomiasis in endemic areas is to avoid contact between the animals and the parasite [9, 10]. With the presence of large permanent water bodies and marshy grazing areas in the Tis Abay district, bovine schistosomiasis might be present in the area. Despite the fact that the biological environment is conducive to the prevalence of infection in the district, no research on bovine schistosomiasis is currently being conducted. As a result, the current study was carried out to ascertain the prevalence and potential risk factors for bovine schistosomiasis in the Tis Abay district, Northern Ethiopia.

## 2. Materials and Methods

### 2.1. Study Area

**Figure 1:**
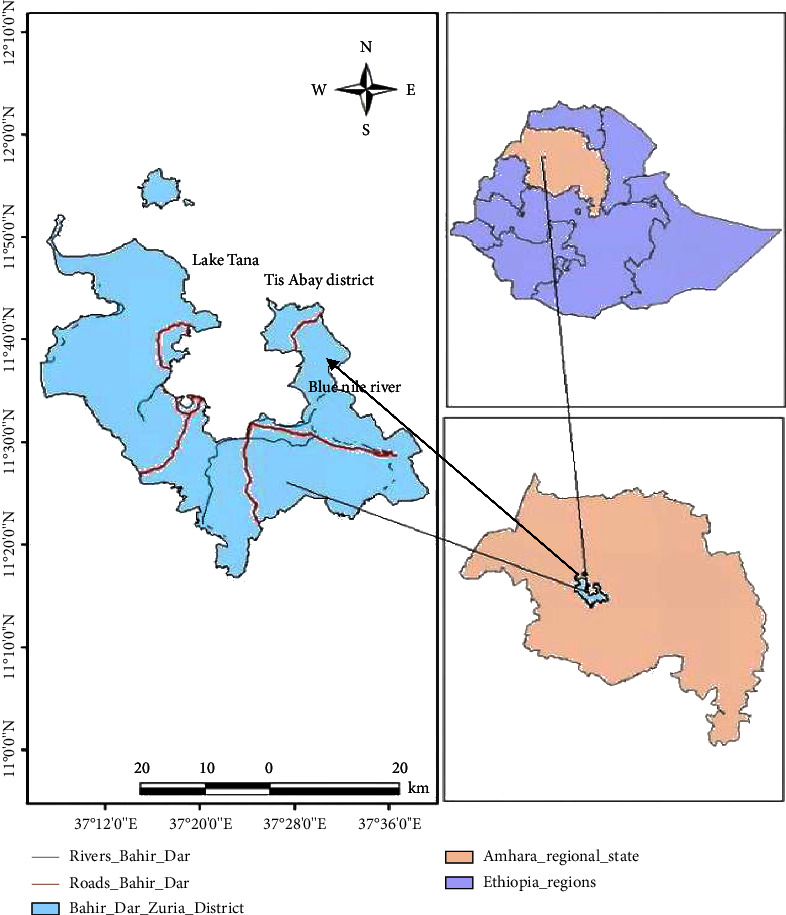
Map of the study area, ArcGIS software, 2020.

The study was conducted from January to August 2021 in selected areas of Tis Abay district (Magi, Dasra, and Yegind Zemocha), Northwest Ethiopia, which is located 595 km from Addis Ababa. This area is bordered by Lake Tana and has an altitude ranging from 1600–1800 meters above sea level. The area has a warm humid climate with an average annual rainfall of 700 mm. The annual temperature of the area ranges from 12.40–27°C. The landscape is marked by the presence of Lake Tana, which drains a watershed of approximately 3,000 km^2^, and areas adjacent to Lake Tana, the Abay River, and the Tikurit River have poor drainage and annual flooding during the dry months (Figure 1). The total cattle population in the western Gojjam zone was 1,800,917 [11].

### 2.2. Study Design and Animals

A cross-sectional study was conducted in cattle of the Tis Abay district to determine the prevalence of bovine schistosomiasis and its associated risk factors. Cattle of different body condition scores, ages, sexes, and origins were included in the study. The age of each animal was estimated using the owner's recorded data and dentition pattern [12]. The body condition of the cattle was categorized into good, medium, and poor depending on the presence of muscle on the different parts of the body. Cattle in “poor/thin” condition (BCS 1–3) are angular and bony with minimal fat over the backbone, ribs, hooks, and pins and with no visible fat around the tail head or brisket. Cattle in the “medium” condition (BCS 4-5) have a good overall appearance with visible hips, although there is some fat over the hooks and pins, and the backbone is no longer visible. Ideal or good conditioned (BCS 6-7) cattle with BCS of 6 or 7 become fleshy, and the ribs are no longer visible with fat around the tail head and brisket [13]. The farm types were classified as intensive, extensive, and semi-intensive (kept indoors based on the farm management system). In the present study, different breed types, namely, local zebu and crossbreed (both crosses of Holstein Friesian with local zebu breed), were included.

### 2.3. Sampling Method and Sample Size Determination

The study animals were selected using a simple random sampling technique from the three selected peasant associations. The study areas were selected based on the total number of cattle populations, the presence of water bodies (either marshy, stagnant, or marshy), and the accessibility of the selected study areas. The sample size of the study was determined using the formula given by Thrusfield [14]. Furthermore, an absolute precision of 5% and a confidence level of 95% were used. The required sample size was determined by considering the previous prevalence of bovine schistosomiasis (13.70%) by Chanie et al. [15] in the Fogera district of Northwest Ethiopia.

(1) $$\begin{array}{c} \frac{n=Z^{2}x\;P\exp\left(1-P\;\exp\right)}{d^{2}}\text{,} \end{array}$$

where *n* = the required sample size, *Z* = critical value of the normal distribution at the required confidence level (1.96), *P*_exp_ = expected prevalence (13.7%), and *d* = desired absolute precision (0.05). Hence, the sample size was calculated to be 181 cattle considering the previous prevalence. However, to increase the precision of the study, the sample size was increased to 384 for fecal sampling.

### 2.4. Fecal Sample Collection and Laboratory Examination

Fecal samples were directly collected from the rectum of cattle found in different farms of the study areas using a gloved hand. The collected samples were preserved in 10% formalin in clean and labeled screw cap universal bottles to prevent the hatching of miracidia before reaching the laboratory, and the samples were examined within 24 h of collection. Then, the samples were placed in an icebox and transported to the veterinary parasitology laboratory of Bahir Dar University.

Approximately 3 g of feces was placed into a centrifuge tube, and 40 ml of water was added and then mixed thoroughly. The suspension was filtered through a tea strainer into another centrifuge tube and left for 15 min. Thereafter, the supernatant was decanted, and the sediment was resuspended. This step was repeated 3 times until the supernatant was clear. Finally, the sediment was transferred with a pipette to a clean slide and observed under a low-power (10x) microscope. The slides were judged positive when oval to spindle-shaped with centrally bulged and terminal spines on one side of an egg were identified [16].

### 2.5. Data Management and Analysis

The data were entered into an MS Excel 2019 spreadsheet, coded, and then analyzed using Stata 13.0 version statistical software program. The prevalence was calculated by dividing the number of positive animals by the total number of animals tested. Logistic regression analyses were conducted using Schistosoma infection as an outcome variable against each of the explanatory variables of the hypothesized risk factors (sex, age, breed, body condition, and management system). The explanatory variables with a *p* value ≤0.25 in univariable analyses were selected for multiple logistic regression analyses. The final multiple logistic regression models were manually built using a forward stepwise selection approach. A variable was considered as a confounder if it changed the coefficient of the significant variables by more than 25%. The multicollinearity of the predictors in the models was also assessed using Kruskal gamma statistics, and those variables whose gamma values ranged between −0.6 and +0.6 were considered in a multivariable logistic regression model. The odds ratio (OR) and its 95% confidence interval (CI) of the variables associated with the outcome variables were calculated from the final multivariate logistic regression models. The Hosmer–Lemeshow goodness of fit test was performed in order to test the model fit the data. A *p* value less than 0.05 was considered statistically significant.

## 3. Results

### 3.1. Prevalence of Schistosomiasis in the Study Area

Out of 384 fecal samples, 50 (13.02%) samples were found to be positive for Schistosoma eggs. The highest prevalence of schistosomiasis was recorded in Dasra (22.4%), followed by Yegind zemocha (15%), and Magi (3.3%). There was a statistically significant (*p* ≤ 0.0001) association between Schistosoma infection and the study area (Table 1).

**Figure 2:**
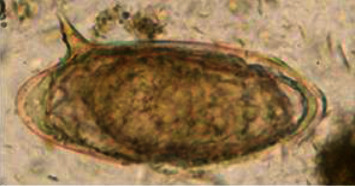
Egg of *Schistosoma bovis* (oval to spindle-shaped with centrally bulged and terminal spines on one side of an egg).

The fecal examination revealed that the egg of Schistosoma bovis was recovered with oval to spindle-shaped with centrally bulged and terminal spines on one side of the egg as indicated in Figure 2.

### 3.2. Role of Risk Factors in the Prevalence of Schistosomiasis

All those independent variables, which were significant in the initial univariable analysis checked for collinearity using Kruskal gamma statistics, and those variables whose gamma value ranged between −0.6 and +0.6 were considered in a multivariable logistic regression model. Accordingly, no collinearity was detected between these variables and was used for multivariable analysis. Thus, study area, breed, body condition, and management system were included in the multivariable model.

The prevalence of bovine schistosomiasis was higher in females (16.9%), crossbred cattle (17.1%), extensively managed cattle (17.9%), and in cattle greater than five years old (23.1%). The odds ratio of bovine Schistosoma infection in the crossbreed was 3.51 (CI; 1.40–8.76) times higher than in local breed cattle. An adjusted odds ratio of Schistosoma infection increase by 3.5 times than it occurs in crude odds ratio while holding the other predictors constant. The odds ratio of bovine Schistosoma infection in poorly conditioned cattle were 0.04 (CI; 0.01–0.16) times higher than in medium-conditioned cattle, while good condition cattle are held constant. The multivariable analysis of risk factors showed that study area, breed, body condition, and management system had significant (*p* < 0.05) effects on the prevalence of bovine schistosomiasis. The Hosmer–Lemeshow goodness of fit test suggested that the model fit the data (ROC curve, receiver operating characteristic curve = 91.28%) and the overall p value of the model is *p* ≤ 0.00001 (Table 2).

## 4. Discussion

The study revealed that 13.02% of the cattle were found to be positive for Schistosoma eggs, which is comparable with the report of Chanie et al. [15], who reported 13.7% in Fogera, Northwestern Ethiopia. This prevalence is relatively lower than a previous study by Wudeneh [17], who reported 16.28% in Fogera Woreda, Northwestern Ethiopia; Yihunie et al. [18], who reported 22.2% in the South Achefer district; Melkamu [19], in Bahr Dar, who reported 24.3%; Habtamu and Mariam [20], who reported 37.3%; Solomon [21], who reported 22.06% in and around Bahr Dar; and Alemseged [22], who reported 27.13% in Dembia. The low prevalence of schistosomiasis reported in our study may be due to sample size, seasonal variation, humidity, management practices, and climate change. However, this result is higher than the report of Belete and Engdaw [23], who reported 10.3% in Fogera Woreda, Northwest Ethiopia; Zelalem [24], who reported 12.5% in Fogera; Almaz [25], who reported 10.93%; Mengistu et al. [26], who reported 10.17%; and Asmare and Samuel [27], who reported 7.6% in Debre Tabor. The differences may be due to the agroecological characteristics of the areas, such as the presence of water bodies (swampy or marshy), irrigation practices, and animal husbandry practices.

There was no statistically significant (*p*=0.524) difference between male and female cattle and the prevalence of bovine schistosomiasis, where a higher prevalence was reported in females (16.9%) than males (8.2%). The current study agrees with the study conducted by Asmare and Samuel [27] and found a higher prevalence in females (33.1%) than males (27.1%). This study disagrees with the previous study of Solomon, [21] in and around Bahir Dar, who reported 29.61% in males and 19.54% in females and Alemseged, [22] in Dembia district 30.7% in males and 23.30% in females. In the present study area, female cattle commonly were grazed in Schistosoma-contaminated pastures and marsh areas, whereas male cattle spent most of their time on farmland plowing activities, which could reduce the risk of exposure to cercaria-infested habitats.

The prevalence of bovine schistosomiasis was significantly (*p*=0.007) higher in crossbred cattle (17.1%) than in local breed cattle (10.5%). Similarly, Lulie and Guadu [28] reported a higher prevalence of bovine schistosomiasis in crossbred cattle (8.3%) than in local breed cattle (7.2%). In contrast, Asmare and Samuel [27] reported a higher prevalence of bovine Schistosoma infection in local breed cattle (24.5%) than in crossbred cattle (18.6%). In the current study area, crossbred cows were kept for milk purposes but were extensively and semi-intensively managed for a long period of time. The management techniques used increased the risk of infection.

The prevalence of bovine schistosomiasis was significantly (*p*=0.006) higher in cattle greater than 5 years old (23.1%) than in cattle aged between 3 and 4 years old (8.1%), while the lowest prevalence was observed in cattle less than 2 years old (4.1%). This is in close agreement with Mengistu et al. [26] and Merawe et al. [29], who stated a significant effect of age on Schistosoma bovis infection in animals. In contrast to the present finding, Alemseged [22] reported a prevalence rate of 17.6% in cattle below 2 years of age, 30.10% in cattle between 2 and 5 years of age, and 27.80% above 5 years of age in Dembia district. In this study, depending on age, cattle greater than 5 years old had the highest prevalence. Animals that graze in swamps all day long and managed extensively have more access to contact with intermediate hosts and increase the risk of Schistosoma infection. The prevalence was lower due to mitigated risk in management practices.

In the current study, the body condition scores of the cattle were significantly (*p*=0.001) associated with the prevalence of bovine schistosomiasis. The highest prevalence was observed in poorly conditioned cattle (37.1%) compared with cattle with moderate body condition (13.8%). In line with this result, Shiferaw and Deressa [30] reported a prevalence of 32.46% in poorly conditioned cattle and 21.42% in cattle compared to medium body conditions. Fromsa et al. [8] in Jimma and Agaro also reported that the prevalence of schistosomiasis was 23.81% in poorly conditioned cattle and 3.92% in medium body-conditioned cattle but not in well-conditioned cattle. This study found that animals with poor body condition scores were more affected than other groups of animals.

The prevalence of bovine schistosomiasis was higher in the extensive management system (17.9%) than in the semi-intensive management (9.6%) and intensive management systems. The difference was statistically significant compared to semi-intensive (9.6%) and intensive treatment (*p*=0.0019). This finding was consistent with Alemseged [22]. Animals in extensive management systems are more exposed to Schistosoma than semi-intensive and intensively reared or reared animals. From this study, it seems that prevalence was higher in extensively managed cattle because the disease transmission required animals to come into contact with swamp snails and cercariae.

The highest prevalence of schistosomiasis was recorded in Dasra (22.4%), followed by Yegind zemocha (15%) and Magi (3.3%). There was a statistically significant (*p* ≤ 0.0001) difference in the prevalence of Schistosoma infection in the study area. The presence of stagnant water, the number of rivers and streams, differences in the spread of the disease due to high humidity in most grazing areas, and the actual fluctuations caused by Dasra were due to the presence of the Abay River in Lake Tana. The Tikurit River forms a favorable environment for intermediate host snails. This makes more favorable conditions for the multiplication of intermediate hosts, hence increasing the chance of Schistosoma infection to occur. Chanie et al. [15] and Abie et al. [31] reported that poorly drained areas with deforested water and acidic soil are often peculiar to *Schistosoma japonicum*. Therefore, schistosomiasis was found in the study area since there was a swamp from the nearest location suitable for snail breeding.

### 4.1. Limitations of the Study

The present study does not determine the intensity of the infection through a quantitative method for a diagnosis such as egg counting and the clinical score of the infected animal. In addition, this research work was conducted with smaller area coverage and in a limited period of the year, which makes it difficult to make causal associations between potential risk factors and bovine schistosomiasis.

## 5. Conclusion

The occurrence of schistosomiasis is closely linked with environmental factors suitable for the development, breeding, and multiplication of the intermediate host (snail) and the parasite itself. Risk factors such as age, body condition score, management, sex, and study sites had a significant effect on the prevalence of schistosomiasis in cattle, but the breed did not show any significant effect. Cattle owners need to be aware of the risk factors for Schistosoma infection in their livestock. In conclusion, there should be regular deworming and veterinary services for cattle in the study area. Farmers should be aware of the risk factors for Schistosoma infection in their animal production. Awareness should be provided to cattle owners about the prevention and control of snails by removing the swampy areas.

## Figures and Tables

**Table 1:** Prevalence of bovine schistosomiasis based on the study area (origin) risk factors.

Study area	No. of examined	No. positive	Prevalence (%)	*χ* ^2^ (*p* value)	95% CI
Dasra	134	30	22.4	39.79 (≤0.0001)	17.6–32.8
Y/zemocha	100	15	15.0		10.2–25.8
Magi	150	5	3.3		3.18–22.58

*χ* ^2^: chi-square; CI: confidence interval.

**Table 2:** Univariable and multivariable logistic regression analysis of risk factors for bovine schistosomiasis.

Factors	Category	No. of examined	No. positive (%)	COR (95% CI)	*p* value	AOR (95% CI)	p value
Study area	Dasra	134	30 (22.4)	8.37 (3.14–22.31)	0.0001	3.47 (1.14–10.61)	0.029
	Y/zemocha	100	15 (15.0)	5.12 (1.79–14.59)	0.002	6.00 (2.11–17.10)	0.001
	Magi	150	5 (3.3)	Ref	Ref	Ref	Ref

Sex	Female	213	36 (16.9)	Ref	Ref	Ref	Ref
	Male	171	14 (8.2)	0.54 (0.29–1.02)	0.059	0.79 (0.38–1.62)	0.524

Breed	Local breed	238	25 (10.5)	Ref	Ref	Ref	Ref
	Crossbreed	146	25 (17.1)	1.60 (0.88–2.91)	0.121	3.51 (1.40–8.76)	0.007

Age	≤2 y	88	4 (4.5)	Ref	Ref	Ref	Ref
	3-4 y	149	12 (8.1)	2.48 (0.68–9.05)	0.169	2.54 (0.63–10.22)	0.187
	≥5 y	147	34 (23.1)	8.85 (2.63–29.76)	0.0001	5.78 (1.64–20.34)	0.006

BCS	Good	155	4 (2.6)	Ref	Ref	Ref	Ref
	Medium	167	23 (13.8)	0.25 (0.056–0.49)	0.0001	0.35 (0.16–0.78)	0.011
	Poor	62	23 (37.1)	0.031 (0.01–0.11)	0.0001	0.04 (0.01–0.16)	0.0001

Management system	Extensive	218	39 (17.9)	Ref	Ref	Ref	Ref
	Semi-intensive	114	11 (9.7)	0.49(0.24–0.99)	0.05	2.24 (1.30–4.18)	0.0019
	Intensive	52	0 (0)	—	—	—	—

*χ* ^2^: chi-square, BCS: body condition score, Ref: variable used as reference, and CI: confidence interval.

## Data Availability

The data used to support the findings of this study are available from the corresponding author upon request.

## References

[1] Ababa A., Central Statistical Agency (2021). Agricultural sample survey on livestock and livestock characteristics (private peasant holdings, Statistical bulletin. *Central Statistical Agency*.

[2] Dermauw V., Dorny P., Braae U. C. (2018). Epidemiology of Taenia saginata taeniosis/cysticercosis: a systematic review of the distribution in southern and eastern Africa. *Parasites & Vectors*.

[3] Yeneneh A., Kebede H., Fentahun T., Chanie M. (2012). Prevalence of cattle flukes infection at andassa livestock research center in north-west of Ethiopia. *Veterinary Research Forum: An International Quarterly Journal*.

[4] Urquhart G., Armour J., Duncan J., Dunn A., Jennings F. (2003). Veterinary parasitology. *Black well science*.

[5] Jejaw A., Zemene E., Alemu Y., Mengistie Z. (2015). High prevalence of Schistosoma mansoni and other intestinal parasites among elementary school children in Southwest Ethiopia: a cross-sectional study. *BMC Public Health*.

[6] Niaz S., Tanveer A., Qureshi A. (2010). Prevalence of schistosomiasis in cows and buffaloes at different sites of Punjab Pakistan and its relation to temperature, relative humidity, rainfall and pan evaporation. *Pakistan Journal of Science*.

[7] Arshad G., Maqbool A., Qamar M., Bukhari S., Hashmi H., Ashraf M. (2011). Epidemiology of schistosomiasis in buffaloes under different managemental conditions in four district of Punjab, Pakistan. *Journal of Animal and Plant Sciences*.

[8] Fromsa A., Meharenet B., Mekibib B. (2011). Major trematode infections of cattle slaughtered at Jimma Municipality Abattoir and the occurrence of the intermediate hosts in selected water bodies of the zone. *Journal of Animal and Veterinary Advances*.

[9] Richter J. (2003). The impact of chemotherapy on morbidity due to schistosomiasis. *Acta Tropica*.

[10] Molla G., Tintagu T., Yasin A., Alemu B., Assen A. A., Tadesse K. (2022). Bovine schistosomiasis in some selected areas of South wollo and oromia zones of Amhara region, north-east Ethiopia. *PLoS One*.

[11] Ababa A., Central Statistics Agency (2019). Federal democratic republic of Ethiopia agricultural sample survey. *Report on livestock and livestock characteristics, Statistical Bulletin*.

[12] Pace J., Wakeman D. (2003). *Determining the Age of Cattle by Their Teeth*.

[13] Nicholson M., Butterworth M. (1986). A guide to body condition scoring of zebu cattle. *International Livestock Center for Africa-ILCA, Addis Ababa, Ethiopia*.

[14] Thrusfield M. (2005). *Veterinary Epidemiology*.

[15] Chanie M., Dejen B., Fentahun T. (2012). Prevalence of cattle schistosomiasis and associated risk factors in Fogera cattle, South gondar zone, Amhara national regional state, Ethiopia. *Journal of Advanced Veterinary Research*.

[16] Elsheikha H. M., Khan H. (2011). *Essentials of Veterinary Parasitology*.

[17] Wudeneh A. (2018). A study on prevalence of bovine schistosomiasis in Fogera woreda, north western of Ethiopia. *Acta Parasitologica Globalis*.

[18] Yihunie A., Urga B., Alebie G. (2019). Prevalence and risk factors of bovine schistosomiasis in Northwestern Ethiopia. *BMC Veterinary Research*.

[19] Melkamu S. (2016). Study on prevalence and associated risk factors of bovine and human schistosomiasis in Bahir Dar and its surrounding areas. *Journal of Animal Research*.

[20] Habtamu A., Mariam S. (2011). Repeated simple sedimentation technique and prevalence of bovine schistosomosis in selected sites of Bahir Dar woreda. *Ethiopian Veterinary Journal*.

[21] Solomon A. (2008). Observation on the prevalence the prevalence of schistosoma bovis infection in Bahir dar area, north Central Ethiopia. *Global Veterinaria*.

[22] Alemseged G. (2010). *Prevalence of Bovine Schistosomiasis in Dembia District, North West Ethiopia*.

[23] Belete M. E., Engdaw T. A. (2015). Study on the prevalence of bovine schistosomiasis in Fogera woreda, north-west of Ethiopia. *European Journal of Biological Sciences*.

[24] Zelalem A. (2010). *Prevalence of Bovine Schistosomiasis in Fogera Woreda, Northwest Ethiopia*.

[25] Almaz H. (2007). *Pathology of Naturally Occurring Schistosoma Infection in Cattle Slaughtered at Bahir Dar Municipal Abattoir North West Ethiopia*.

[26] Mengistu S., Tewodros F., Mersha C. (2012). Prevalence of bovine schistosomiasis in Fogera district, South gondar zone, Amhara national regional state, northwest Ethiopia. *Global Veterinaria*.

[27] Asmare G., Samuel D. (2015). Prevalence of bovine fasciolosis and its associated risk factor in and around dangila district, awi administration zone, northwestern Ethiopia. *European Journal of Biological Sciences*.

[28] Lulie B., Guadu T. (2014). Bovine schistosomiasis: a threat in public health perspective in Bahir Dar town, northwest Ethiopia. *Acta Parasitologica Globalis*.

[29] Merawe M., Kassaw Amssalu Y. H., Afera B. (2014). Intestinal schistosomiasis of bovine and ovine in Fogera district, South gonder zone, Amhara national regional state, Ethiopia. *Young*.

[30] Shiferaw M. B., Deressa F. B. (2017). Prevalence and associated risk factors of bovine schistosomiasis in and around bakko town, west shoa zone, oromia, Ethiopia. *Global Journal of Science Frontier Research: D Agriculture and Veterinary*.

[31] Abie S., Alemu S., Derso S., Demessie Y., Yibarek D. (2016). A cross sectional study on the prevalence and possible risk factors of bovine schistosomiasis in and around Bahir dar town, northwest Ethiopia. *European Journal of Biological Sciences*.

